# Comparative Genomics and Integrated Network Approach Unveiled Undirected Phylogeny Patterns, Co-mutational Hot Spots, Functional Cross Talk, and Regulatory Interactions in SARS-CoV-2

**DOI:** 10.1128/mSystems.00030-21

**Published:** 2021-02-23

**Authors:** Vipin Gupta, Shazia Haider, Mansi Verma, Nirjara Singhvi, Kalaisaran Ponnusamy, Md. Zubbair Malik, Helianthous Verma, Roshan Kumar, Utkarsh Sood, Princy Hira, Shiva Satija, Yogendra Singh, Rup Lal

**Affiliations:** a PhiXGen Private Limited, Gurugram, Haryana, India; b Jaypee Institute of Information Technology, Noida, Uttar Pradesh, India; c Department of Zoology, Sri Venkateswara College, University of Delhi, New Delhi, India; d Department of Zoology, University of Delhi, Delhi, India; e School of Biotechnology, Jawaharlal Nehru University, New Delhi, India; f School of Computational and Integrative Sciences, Jawaharlal Nehru University, New Delhi, India; g Department of Zoology, Ramjas College, University of Delhi, Delhi, India; h P.G. Department of Zoology, Magadh University, Bodh Gaya, Bihar, India; i The Energy and Resources Institute, New Delhi, India; Oxford Nanopore Technologies

**Keywords:** comparative genomics, phylogenomics, phylogeny, SARS-CoV-2, mutational studies, structural biology

## Abstract

The severe acute respiratory syndrome coronavirus 2 (SARS-CoV-2) pandemic has resulted in 92 million cases in a span of 1 year. The study focuses on understanding population-specific variations attributing its high rate of infections in specific geographical regions particularly in the United States. Rigorous phylogenomic network analysis of complete SARS-CoV-2 genomes (245) inferred five central clades named a (ancestral), b, c, d, and e (subtypes e1 and e2). Clade d and subclade e2 were found exclusively comprised of U.S. strains. Clades were distinguished by 10 co-mutational combinations in Nsp3, ORF8, Nsp13, S, Nsp12, Nsp2, and Nsp6. Our analysis revealed that only 67.46% of single nucleotide polymorphism (SNP) mutations were at the amino acid level. T1103P mutation in Nsp3 was predicted to increase protein stability in 238 strains except for 6 strains which were marked as ancestral type, whereas co-mutation (P409L and Y446C) in Nsp13 were found in 64 genomes from the United States highlighting its 100% co-occurrence. Docking highlighted mutation (D614G) caused reduction in binding of spike proteins with angiotensin-converting enzyme 2 (ACE2), but it also showed better interaction with the TMPRSS2 receptor contributing to high transmissibility among U.S. strains. We also found host proteins, MYO5A, MYO5B, and MYO5C, that had maximum interaction with viral proteins (nucleocapsid [N], spike [S], and membrane [M] proteins). Thus, blocking the internalization pathway by inhibiting MYO5 proteins which could be an effective target for coronavirus disease 2019 (COVID-19) treatment. The functional annotations of the host-pathogen interaction (HPI) network were found to be closely associated with hypoxia and thrombotic conditions, confirming the vulnerability and severity of infection. We also screened CpG islands in Nsp1 and N conferring the ability of SARS-CoV-2 to enter and trigger zinc antiviral protein (ZAP) activity inside the host cell.

**IMPORTANCE** In the current study, we presented a global view of mutational pattern observed in SARS-CoV-2 virus transmission. This provided a who-infect-whom geographical model since the early pandemic. This is hitherto the most comprehensive comparative genomics analysis of full-length genomes for co-mutations at different geographical regions especially in U.S. strains. Compositional structural biology results suggested that mutations have a balance of opposing forces affecting pathogenicity suggesting that only a few mutations are effective at the translation level. Novel HPI analysis and CpG predictions elucidate the proof of concept of hypoxia and thrombotic conditions in several patients. Thus, the current study focuses the understanding of population-specific variations attributing a high rate of SARS-CoV-2 infections in specific geographical regions which may eventually be vital for the most severely affected countries and regions for sharp development of custom-made vindication strategies.

## INTRODUCTION

Severe acute respiratory syndrome coronavirus 2 (SARS-CoV-2) is a single-stranded RNA virus with a genome size ranging from 29.8 kb to 29.9 kb (1). Most countries are facing second waves and are on the verge of the next wave. So far more than 18 million deaths and 800 million active cases have been reported worldwide (https://www.worldometers.info/coronavirus/). The genomic repertoire of SARS-CoV-2 comprises of 10 open reading frames (ORFs) encoding 27 proteins (2). ORF1ab encodes 16 nonstructural proteins (Nsp), whereas structural proteins include spike (S), envelope (E), membrane (M), and nucleocapsid (N) proteins (3, 4). In addition, the genome of SARS-CoV-2 is comprised of ORF3a, ORF6, ORF7a, ORF7b, ORF8, and ORF9 genes encoding six accessory proteins, flanked by 5′ and 3′ untranslated regions (UTRs) (1). In our previous study (5), a higher mutational rate in the genomes from different geographical locations around the world by accumulation of single nucleotide polymorphisms (SNPs) was reported. Even during these early stages of the global pandemic, genomic surveillance has been used to differentiate circulating strains into distinct, geographically based lineages (6). However, the ongoing analysis of this global data set suggests no consolidated significant links between SARS-CoV-2 genome sequence variability, virus transmissibility and disease severity.

Although there are several studies that have appeared ever since the emergence of SARS-Cov-2 (7, 8) and it has been reflected that the mutations at both the genomic and protein level are in a “Hormonical Orchestra” (9) that drives the evolutionary changes, demanding a detailed study of SARS-CoV-2 mutations to understand its successful invasion and infection. To unveil this, we rendered and screened 18,775 genomes of SARS-CoV-2 and selected 245 genomic sequences deciphering the phylogenetic relationships, tracing them to SNPs at nucleotide and amino acid variation (AAV) levels and performing structural remodeling. We specifically focused on the evolutionary relationships among the strains predicting Nsp3 as a mutational hot spot for SARS-CoV-2. We extended the study to understand the mechanism of host immunity evasion by host-pathogen interaction (HPI) and confirming their interactions with host proteins by docking studies. We identified sparsely distributed hubs which may interfere and control network stability as well as other communities/modules. This indicated the affinity to attract a large number of low-degree nodes toward each hub, which is a strong evidence of controlling the topological properties of the network by these few hubs (10). We also analyzed the transfer of genomic SNPs to amino acid levels and associations of CpG dinucleotides contributing toward the pathogenicity of SARS-CoV-2, since the CpG islands have always been linked with epigenetic regulation and act as the hot spots for methylation in the case of viruses (11–13). However, for RNA viral genomes, CpG nucleotides are the targets for zinc antiviral protein (ZAP), a major factor of mammalian interferon-mediated immune response (14, 15). Here also, the conservancy found in possession of CpG dinucleotides towards the extremities of all the genomes considered in the present analysis indicate their importance in evading host immunity.

## RESULTS AND DISCUSSION

### Phylogenetic relationship between different SARS-CoV-2 strains.

**FIG 1:**
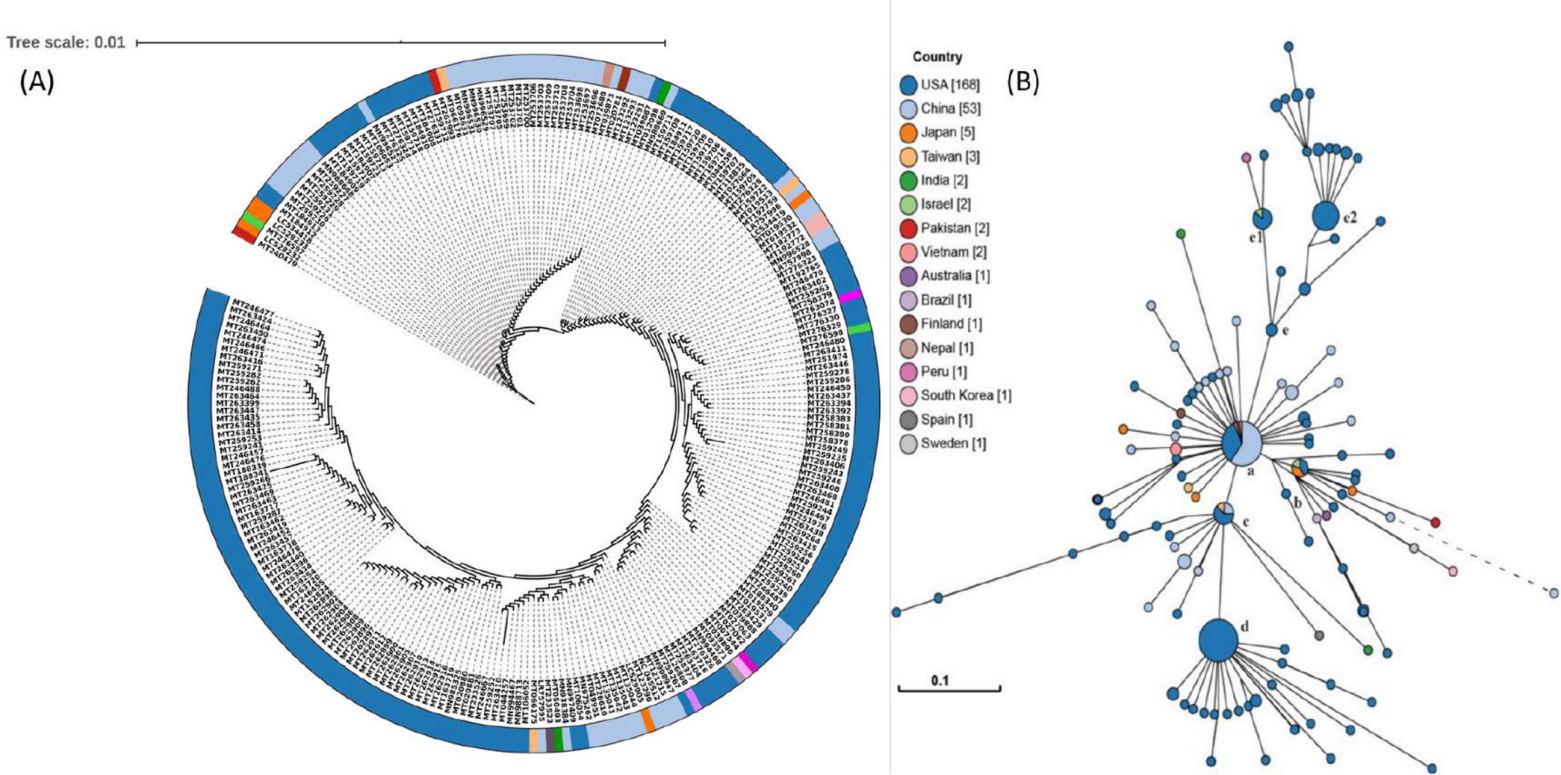
Phylogenetic network of 245 SARS-CoV-2 genomes. (A) Nucleotide-based phylogenetic analysis of SARS-CoV-2 isolates using the maximum likelihood method based on the Tamura-Nei model. (B) Amino acid-based phylogenomic analysis. Circle areas are proportional to the number of taxa. The map is diverged into five major clades (clades a to e) representing variation in the genomes at the amino acid level. The colored circle represents the country of origin of each isolate.

In our previous study, we reported a mosaic pattern of phylogenetic clustering of 95 genomes of SARS-COV-2 isolated from different geographical locations (5). Strains belonging to one country were found clustered with strains from distant countries, but not with strains from the neighboring country. Taking clues from this study, we constructed phylogenetic relatedness of 245 strains of SARS-COV-2 from the United States, China, and several other countries, including Spain, Vietnam, Peru, Finland, and Pakistan, and unravel the significant association of evolutionary patterns among SARS-CoV-2 based on their geographical locations predicting their mosaic phylogenetic arrangements. It was found that most strains from the United States were clustered together, but comparatively high divergence was found in strains isolated from China and Japan. Japanese strains were found to be scattered and formed clusters with strains from the United States, Pakistan, Vietnam, Taiwan, and China. Even a smaller number of genome sequences from Japan, Vietnam, and Peru revealed a highly scattered pattern, and close associations with U.S. and Chinese strains were revealed. Strains were reported from patients from Taiwan (MT192759), Australia (MT007544), South Korea (MT039890), Nepal (MT072688), and Vietnam (MT192773, MT192772) who had traveled to Wuhan, China (16). However, a strain from Pakistan (MT240479) which clustered with the Japanese strains was found to be isolated from a patient having a travel history to Iran. Indian strains (MT050439 and MT012098) that were isolated from patients who travelled from Dubai, clustered with Chinese strains. Later, reports confirmed many cases of SARS-CoV-2 in Dubai from China (https://www.newsbytesapp.com/timeline/India/58169/271167/coronavirus-2-positive-cases-detected-in-delhi-telangana). Thus, a clear landscape of phylogenetic relationships could be obtained reflecting mosaic clustering patterns in accordance with the travel history of patients (Fig. 1A). However, results were in contradiction with the genomic analysis of SARS-COV-2 by Forster et al. (6) where they predicted the linear/directive evolution from ancestral node a to nodes b and c. We report here both divergent (from ancestral node a to b, c and e) and directive (node c to d) evolution among the SARS-CoV-2 strains (Fig. 1B).

Since genome-based phylogeny did not highlight the amino acid level changes, to ascertain the variations among the SARS-CoV-2 strains at the protein level, we constructed whole-proteome alignment-based phylogeny, clustered the 245 strains into five major clades, clades a to e (Fig. 1B). The first cluster, clade a had maximum nodes (46), including the reference node, and strains from Nepal (MT072688), Pakistan (MT262993), Taiwan (MT192759) along with 15 strains from the United States and 27 strains from China. It also had the mutated daughter nodes radiating outwards, belonging to China, Finland (MT020781), India (MT012098), Japan (LC534419 and LC529905), Taiwan (MT066176), Vietnam (MT192772-3), Brazil (MT126808), Australia (MT007544), South Korea (MT039890), and Sweden (MT093571) along with seven U.S. strains (Fig. 1B). This clade represented the ancestral node, as it harbored the oldest known SARS-CoV-2 strain from China and laid the foundation for the rest of the mutated daughter strains worldwide, marking the onset of the divergence in SARS-CoV-2. Three significantly diverged network nodes originated from the ancestral clade a and were marked as clades b, c, and e (Fig. 1B). For clade b, the central node included only four strains in which two were from the United States (MT184912 and MT276328) and one each from Israel (MT276597) and Japan (LC528233). Its major descended radiating nodes belonged to Japan (LC528232 and LC534418), Pakistan (MT240479), United States (MT184913, MT184910, and MN997409), and China (MT049951 and MT226610). It was observed that one of the Chinese strains in clade b (MT226610) had the longest branch length, making the strain very distinct (harboring 25 other mutations) by showing an exceptionally high rate of evolution. In the clade c lineage, the small central node was comprised of Taiwan (MT066175), U.S. (MT246667, MT233526, MT020881, MT985325, and MT020880) and Chinese (MN938384 and LR757995) strains. Interestingly, one strain each from Spain (MT233523) and India (MT050493) were also found radiating as daughter nodes from the central one. The clade d lineage, which originated from the clade c lineage, consisted only of U.S. strains both in central nodes and radiations. Importantly, two strains (MT263416 and MT246471) were found most divergent with varied mutations, suggesting the high rate of evolution among U.S. strains, which might be linked to the high pathogenicity among the strains. Clade e bifurcated into two subclades (e1 and e2) by a significant set of mutations. Subclade 1 includes six strains from the United States, one from Israel (MT276598) with radiating nodes from Peru (MT263074) and the United States (MT276327), whereas subclade 2 had 32 strains belonging to the United States. The effects of amino acid mutations were further checked on another subset of 12,299 SARS-CoV-2 genomes (screened from 18,775) for validation. The random explosion of evolutionary clades were seen (see Fig. S1 in the supplemental material). There were other nodes progressing from e (e1-e2) to f (exclusive U.S. strains), g (g1), h, i, and j (exclusive Australian strains), and k subclades. This divergence supported the random evolution of SARS-CoV-2 suggesting network expansion in multiple clades contradicting the earlier directed evolution proposed by Forster et al. (6). Also, the mutational counts (see Data Set S3 in the supplemental material) observed by 12,299 genomes were almost similar to those identified in 245 representative genomes (Fig. S1). Thus, formation of five major evolutionary clades and subclades based on the amino acid phylogeny needs attention for identifying the assessment of divergence among SARS-CoV-2 strains.

10.1128/mSystems.00030-21.1 FIG S1 AAV-based phylogenetic map of 12,299 SARS-CoV-2 genomes. The formation of each clade is well correlated with the mutational combinations produced by nine different mutations. The green clade is represented by the 245 genomes, and the addition of ORF7, N, nsp2, Orf3a mutation to clade 1 resulted in delineation of other combinations to form clades f to j. Download FIG S1, JPG file, 0.2 MB. Copyright © 2021 Gupta et al. 2021 Gupta et al. https://creativecommons.org/licenses/by/4.0/ This content is distributed under the terms of the Creative Commons Attribution 4.0 International license.

### Genotyping and variation estimation.

**FIG 2:**
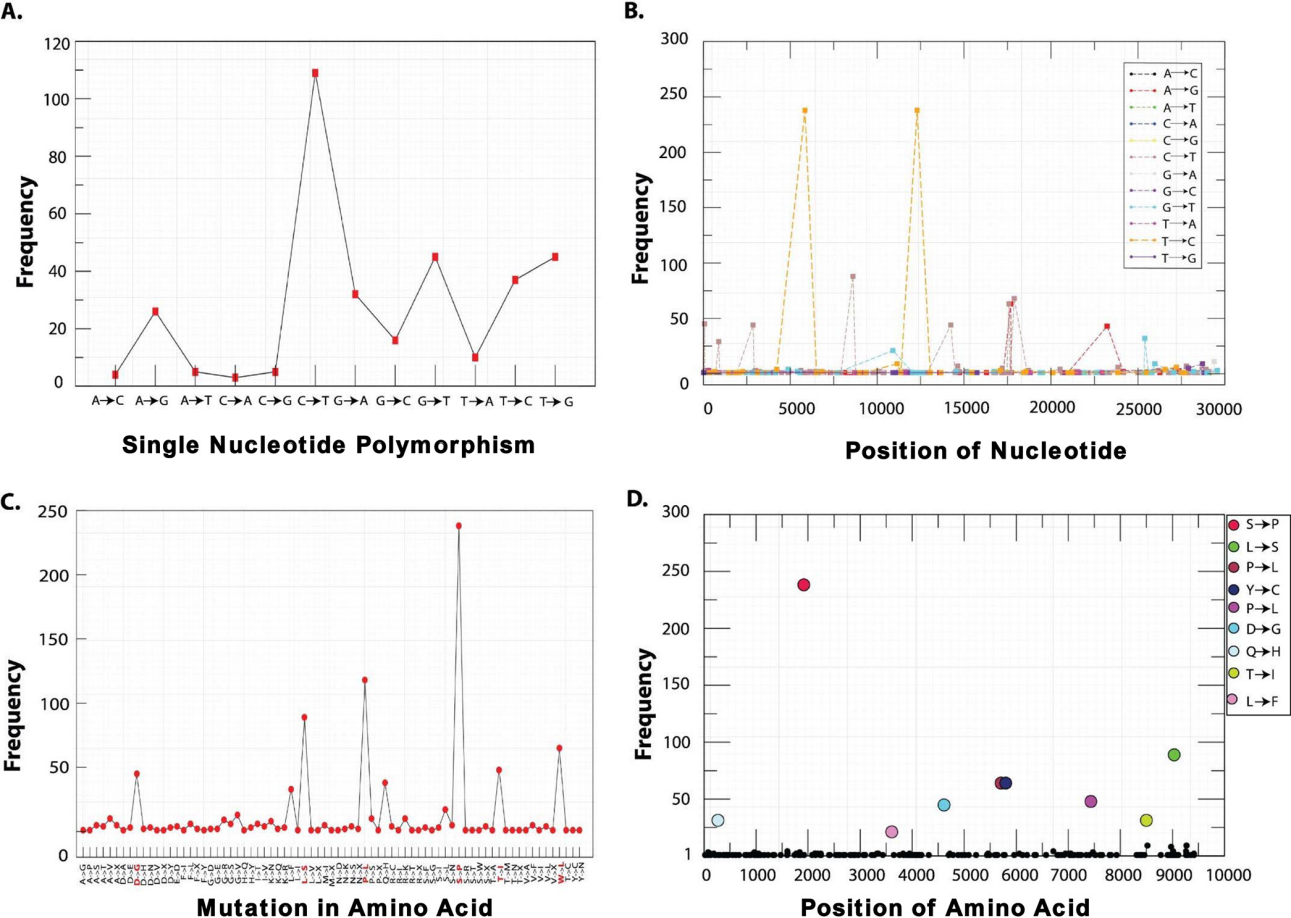
Distribution of SNP (A and B) and AAV (C and D) mutations of SARS-CoV-2 isolates from the globe. (A) Frequency-based plot of 12 possible SNP mutations across 245 genomes. (B) Frequencies of the single SNP mutations with locations on the genome. (C) AAV-based mutations across the genomes. (D) Top 9 AAV mutations holding highest frequencies among 245 genomes and their respective positions. The nucleotide and amino acid positions are based on the reference genome of SARS-CoV-2.

To understand the implication of mosaic pattern of transmissions and evolutionary lineage clustering (clades a to e), we studied the SNP genotyping from the 245 genome sequences as mutation counts along with their frequency at specific genomic locations. Mutational changes at protein/amino acid levels were also weighed by assessing amino acid variation (AAV). Interpolations of the SNP/AAV data were made by assessing their frequency, genomic positions, and type of SNPs/AAVs (Fig. 2B) and highlighted a large mutational diversity among the virus isolates. We identified a total of 12 SNP types (A→G, A→C, A→T, C→A, C→G, C→T, G→A, G→C, G→T, T→A, T→C, and T→G) accounting for mutations at 297 genomic locations (Fig. 2A and B). The overall pattern of SNPs suggested C→T transition as the most common mutation in the entire genomic sets (Fig. 2A); however, the highest frequency was recorded for T→C transitions (Fig. 2B). Based on the genomic arbitrators SNP frequencies, we analyzed 14 major locations inside the genomes of SARS-CoV-2 for potential mutation generating different allelic forms for genes (Table 1). The SNP of C→T was first observed at position 67 in the 5′ UTR region of leader sequence with a frequency of 45 followed by Nsp2 at two locations (885 and 2863) with the frequencies of 29 and 44, respectively. Nsp3/PL-PRO and Nsp8 marked the highest frequency of 238 SNP counts of T→C at positions 5852 and 12299. Another T→C SNP was observed in ORF8 with frequency of 88 at position 27973. The C→T SNP transformation was found in Nsp4 and Nsp12 with the frequency of 88 and 44 at positions 8608 and 14234, respectively. Nonstructural protein Nsp13 was strangely found to harbor two different SNPs (C→T and A→G) at three different locations (positions 17573, 17684, and 17886; Fig. 2B) with relatively high frequencies of 68, 63, and 63, respectively. A→G SNP conversion in S (spike) protein was found with a frequency of 43. A low SNP count of G→T transitions were falling in the ORF3a and Nsp6 with frequency of 32 and 21, respectively (Table 1). However, all SNP counts do not reflect the change at protein level and therefore must be estimated at the translation levels for their significant effect. A total of 297 genomic locations harbored SNPs, but their corresponding AAVs were found only in 200 genomic locations accounting for 67.34% conversion efficiency. Out of 14 high-frequency SNPs, only 9 mutations (Nsp2 [T85I], Nsp3 [S1103P], Nsp6 [L37F], Nsp12 [P324L], Nsp13 [P409L and Y446C], S [D614G], Orf3a [Q577H], and Orf8 [L84S]) were found to be reflected at the protein level with the highest frequency of 238 in Nsp3 (Table 1).

**TABLE 1:** Common SNP and AAV mutations occurring in SARS CoV-2 genomes^*a*^

CDS	Point mutation	Position	Frequency	Amino acid-R	Variant	Position	Frequency
5′UTR	C→T	67	45				
Nsp2	C→T	885	29	T	I	85	31
Nsp2	C→T	2863	44				
Nsp3/PL-PRO	T→C	5852	238	S	P	1103	238
Nsp4	C→T	8608	88				
Nsp6	G→T	10909	21	L	F	37	21
Nsp8	T→C	12299	238				
Nsp12 (RdRp)	C→T	14234	44	P	L	324	46
Nsp13 (Hel)	C→T	17573	63	P	L	409	64
Nsp13 (Hel)	A→G	17684	63	Y	C	446	64
Nsp13 (Hel)	C→T	17886	68				
S	A→G	23232	43	D	G	614	45
Orf3a	G→T	25392	32	Q	H	57	34
Orf8	T→C	27973	88	L	S	84	89

a CDS, coding sequence; Amino acid-R, amino acid residue.

These mutated proteins are known to play various regulatory roles, and therefore, mutations at amino acid level can modulate their catalytic activity drastically. Specifically, Nsp3 is the largest and essential component of replication complex in the SARS-CoV-2 genome (18), and along with Nsp2, it forms a transcriptional complex in the endosome of the infected host cell (19). Nsp6 is a multiple-spanning transmembrane protein located into the endoplasmic reticulum where they induce autophagosomes via an omegasome intermediate (20). Interestingly, the mutation of L37F caused stiffness in the secondary structure of Nsp6 and leads to low stability of the protein structure as observed in most recent strains isolated from Asia, America, Oceania, and Europe (17). Nsp12 and Nsp13 are the key replicative enzymes, which require Nsp6, Nsp7, and Nsp10 as cofactors. Nsp12, a RNA-dependent RNA polymerase (RdRp) with the presence of the bulkier leucine side chain at position 324, is likely to create a greater stringency for base pairing to the templating nucleotide, thus modulating polymerase fidelity (21). Nsp13 contains a helicase domain, allowing efficient strand separation of extended regions of double-stranded RNA and DNA (22). Dual mutations in Nsp13 were reported with profound effect on its activity specifically in the Pacific Northwest of the United States (23). The P409L mutation leads to increased affinity of helicase RNA interaction, whereas Y446C is a destabilizing mutation increasing the molecular flexibility and leading to decreased affinity of helicase binding with RNA (24). Therefore, both the mutations were antagonistic in nature. Thus, ORF1ab polyprotein of SARS-CoV-2 encompasses mutational spectra where signature mutations for Nsp2, Nsp3, Nsp6, Nsp12, and Nsp13 have been predicted.

Amino acid mutations in structural proteins S, ORF3a, and ORF8 have also been observed with varied frequencies of 45, 34, and 89, respectively. The mutation in spike protein (D614G) has been reported to outcompete other preexisting subtypes, including the ancestral one. This mutation generates an additional serine protease (elastase) cleavage site in the spike protein (25) which is discussed in more details in later sections. The ORF3a mutation (Q57H) is located near tumor necrosis factor (TNF) receptor-associated factor 3 (TRAF-3) regions and has been reported as molecular difference marker in many genomes, including Indian SARS-CoV-2 genomes (26) for their delineation. Mutation in ORF8 sequence (L84S) was found conserved (27); therefore, to predict its effect, it was critical to examine its biological function in SARS-CoV-2 interaction with human proteins.

Our results showed that the mutations (SNPs and AAV) in the virus were not uniformly distributed. Genotyping study annotated few mutations in the SARS-CoV-2 genomes at certain specific locations with high frequency predicting their high selective pressure. Thus, mutations can be predicted as location specific but not type specific by SNP count. Highly frequent AAV might be associated with the changes in transmissibility and virulence behavior of the SARS-CoV-2. Therefore, high-frequency AAV mutations in spike protein, RdRp, helicase, and ORF3a are important factors to consider while developing vaccines against the fast-evolving strains of SARS-CoV-2.

### Prevalence of co-mutation in SARS-COV-2 evolution.

Interestingly, we observed co-mutations in Nsp13 at positions 446 (Nsp13_1) and 409 (Nsp13_2) that were prevalent in common 64 genomes, all belonging to the United States. The AAV reported above (Table 1) were further analyzed and found occurring in 10 different permutations varying from single to multiple mutated protein combinations. Complete details of these co-mutations combinations are given in Table 2 and Data Set S2. These co-mutations were mapped over the divergent phylogeny for indicating the evolutionary divergence among the 245 strains. The phylogram (Fig. 1B) showed clear divergence of strains from the parent strain due to accumulation of mutations at different levels of human-to-human transmission. We found co-mutations in Nsp3, ORF8, Nsp13, S, Nsp12, Nsp2, and Nsp6 were responsible for the above divergence.

**TABLE 2:** Co-mutation combinations and genomic locations identified in different proteins of SARS-CoV-2

Variation(s)	(Co)mutation(s)	No. of mutated proteins	No. of descendants
S→P	Nsp3	1	87
Y→C/P→L/S→P/L→S	Nsp13_1/Nsp13_2/Nsp3/ORF8	4	62
S→P/L→S	Nsp3-ORF8	1	22
P→L/D→G/Q→H/S→P/T→I	nsp12/S/ORF3a/Nsp3/Nsp2	5	30
P→L/Q→H/S→P/T→I	Nsp12/ORF3a/Nsp3/Nsp2	4	1
P→L/D→G/Q→H/S→P	Nsp12/S/ORF3a/Nsp3	4	3
L→F/S→P	Nsp6/Nsp3	2	16
L→F/S→P/L→S	Nsp6/Nsp3/ORF8	3	3
Y→C/P→L/L→F/S→P/L→S	Nsp13_1/Nsp13_2/Nsp6/Nsp3/ORF8	5	2
P→L/D→G/S→P	Nsp12/S/Nsp3	3	12

**FIG 3:**
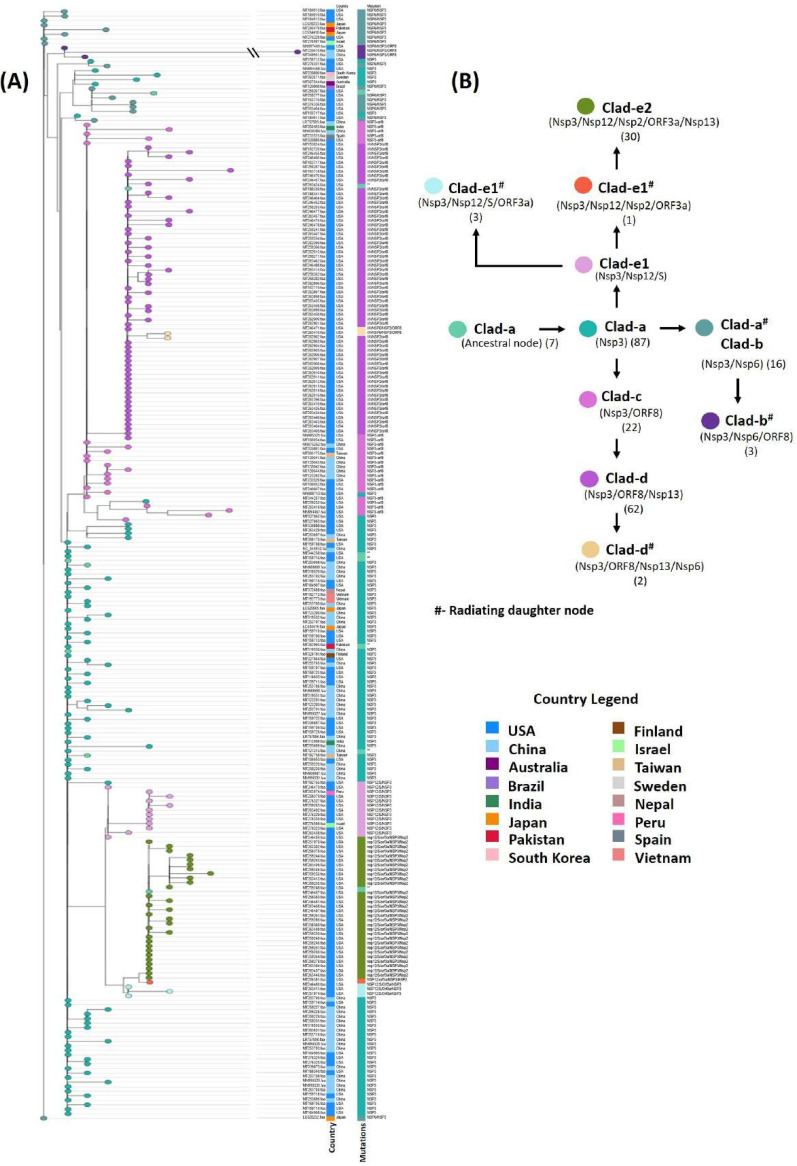
AAV-based phylogenetic map of 245 SARS-CoV-2 genomes. Node color represents co-mutational combinations. The formation of each clade is well correlated with the mutational combinations (*n* = 10). Clad-e2, clade e2.

These co-mutations were found linked with lineage clades a to e, highlighting their prevalence of delineation among them (Fig. 1B). In clade a, 40 genomes harbored mutations at only Nsp3 protein, while six isolates belonging to the United States (MT262993, MT044258, MT159716, MT259248, and MT259267) and Pakistan (MT263424) showed no mutation, confirming that their lineage was the same as that of the reference/ancestral genome from China. The presence of the Nsp3 mutation (S1103P) in 238 strains underlined the origin of mutation from the reference strain highlighting the first mutation- induced divergence in SARS-CoV-2 strains. Therefore, Nsp3 was marked as the first mutational hot spot for accumulating amino acid mutations in SARS-CoV-2. Strains from Brazil (MT126808) and the United States (MT276331) form the descendant from clade a harboring Nsp3/Nsp6 as the first mutational combination directing the common evolutionary lineages. Clade b had an additional mutation of ORF8 along with Nsp3 and Nsp6 with three descendant strains from the United States and China. We observed the most distant Chinese strain (MT226610) clustered in clade b, and it harbored additional 25 AAV, making it the highly pathogenic strain in the network (Fig. 1B). Clade c descended from clade a had a different set of co-mutations with Nsp3-ORF8 proteins, while clade d descended further from clade c had two mutations in Nsp13 (P409L/Y446C) in addition to Nsp3/ORF8 proteins. Two strains from the United States in the cluster radiating from clade d harbored an additional Nsp6 mutation, making them more divergent with scope for further possible evolution. The next subclade, e1, was found to possess another new set of co-mutations of Nsp3/S/Nsp12. The highest number of co-mutations was found in subclade e2 with the Nsp3/Nsp2/Nsp12/S/ORF3a combination prevalent in 30 genomes belonging to the United States, predicting these genomes as an active carrier of evolutionary force for SARS-CoV-2 divergence (Fig. 3). In future, addition of more and more genome may indicate the evolutionary relationships among these co-mutations. Our result suggested that co-mutations are the major evolutionary force that drives the pathogenicity among the different geographical isolated strains and may be responsible for the higher and lower degrees of virulence among these strains.

### Assessment of mutations in SARS-CoV-2 proteins.

**FIG 4:**
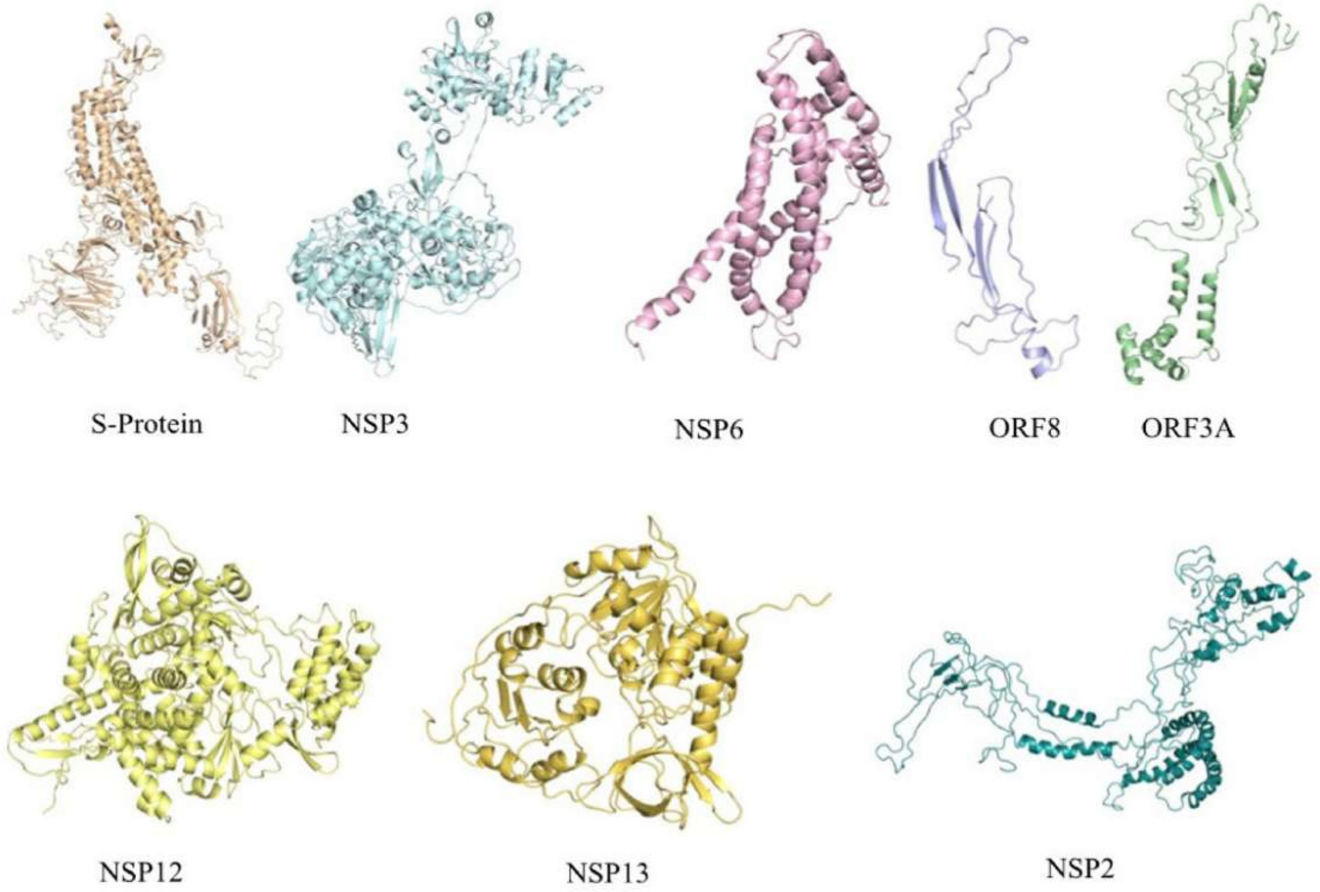
3D structure prediction of SARS-CoV-2 proteins harboring mutations at different locations to check for its stability in the cell. The structures were predicted using SwissModel and Phyre2 servers.

Amino acid variations were predicted in eight (Nsp2, Nsp3, Nsp6, Nsp12, Nsp13, S, Orf3a, and Orf8) SARS-CoV-2 proteins (Table 1). To identify their potential functional role, we carried out the structural analysis of the proteins. Pairwise sequence alignment of wild-type and mutant proteins provided the exact location and changes in amino acids. The GMQE (Global Model Quality Estimation) and QMEAN (Qualitative Model Energy ANalysis) values ranged from 0.45 to 0.72 and −1.43 to −2.81, respectively. The sequence identity ranged from 34% to 99%, which suggested that models were constructed with high value of confidence (Fig. 4). The I-Mutant DDG tool predicts whether a mutation can largely destabilize the protein (ΔΔG < −0.5 Kcal/mol), largely stabilize (ΔΔG > 0.5 Kcal/mol), or have a weak effect (−0.5 ≤ ΔΔG ≤ 0.5 kcal/mol). The protein stability analysis showed that all the identified mutations decreased the stability of seven proteins (Nsp2, Nsp6, Nsp12, Nsp13, S, Orf3a, and Orf8) except Nsp3 (T1103P) which was predicted to increase protein stability (Fig. 4). Further, to explore the role of mutations in SARS-CoV-2 proteins, we carried out HOPE analysis. A D614G mutation in the S-protein could disturb the rigidity of the protein, and due to glycine, hydrophobicity will affect the intra-hydrogen bond formation with G594. In ORF8 and Nsp3, the mutation location was not conserved; hence, it did not affect or damage the protein function. The mutation (P409L) in Nsp13 was present in the RNA virus helicase C-terminal domain. Since proline is a very rigid amino acid and therefore induces a particular backbone conformation that might be required at this position, this mutation could disturb the domain and abolishd its function. Mutation L37F (Nsp6) and T85I (Nsp2) were also highly conserved and thus could profoundly damage the function of the respective protein. The P324L (Nsp12) mutation was in the RNA binding domain located on the surface of the protein; modification of this residue could disturb interactions with other molecules or other parts of the protein. Conclusively, the Nsp3 mutation, which appeared in all co-mutation combinations, contributed to increased protein stability among 238 strains could be assigned to their increased pathogenicity. Thus, we attempted to highlight the effects of these mutations in host-pathogen interactions.

### Modeling of host-pathogen interaction network and its functional analysis.

**FIG 5:**
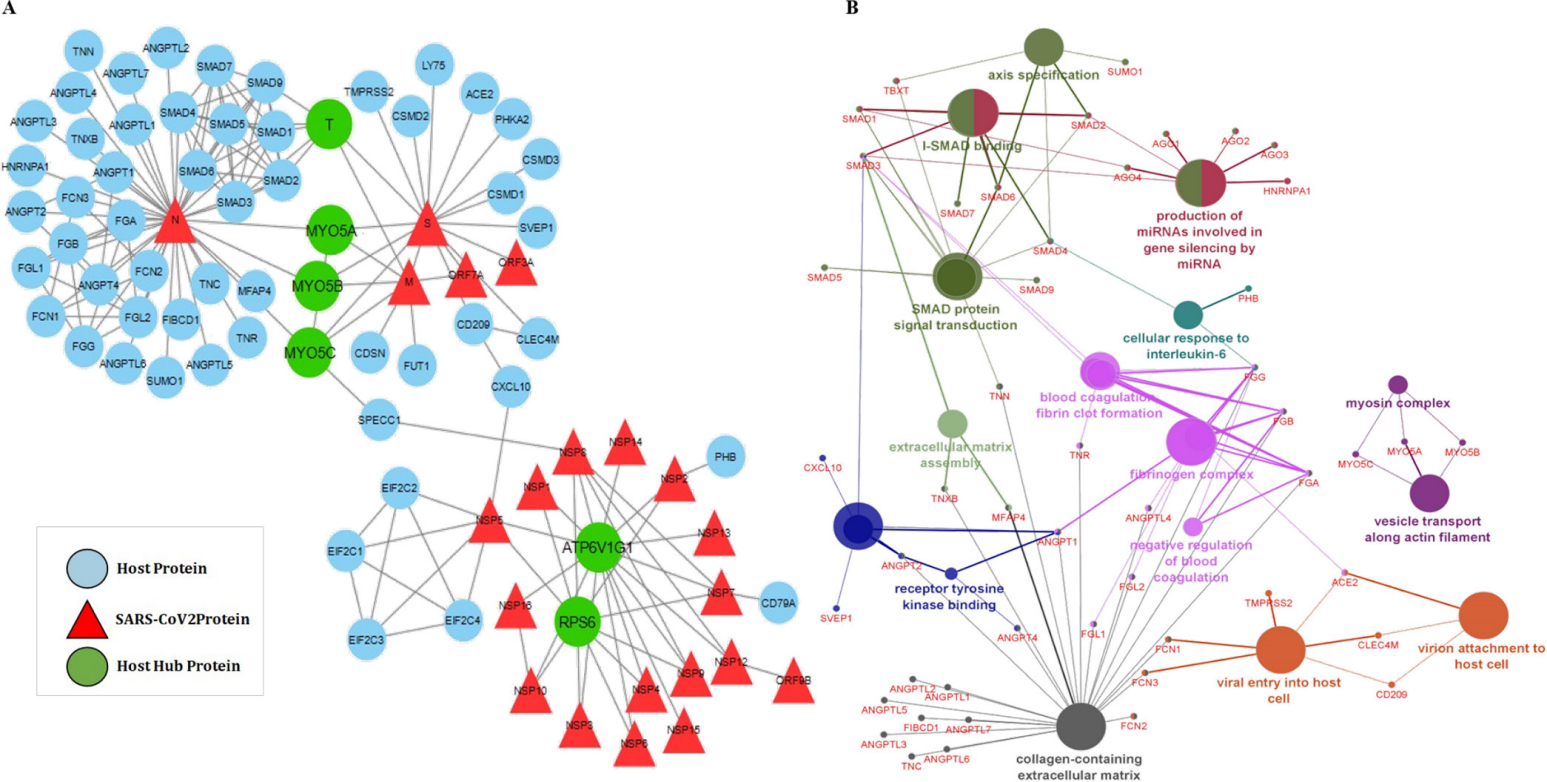
(A) Host-pathogen interaction of SARS-CoV-2 and human proteins. Nodes represent proteins, while lines/edges represent interaction. Triangles (red) represent viral proteins found to be directly interacting with the human proteins (blue). The hubs (MYO5A, MYO5B, MYO5C, T, RPS6, and ATP6V1G1) (green) were found interacting with maximum viral proteins. (B) Gene ontology (GO) analysis was performed for host proteins using the ClueGo Cytoscape app against database KEGG, the Gene Ontology—biological function database, and Reactome pathways. ClueGo parameters were set as follows: Go Term Fusion selected; *P* values of ≤0.05; GO tree interval, all levels; kappa score of 0.42.

The HPI network of SARS-CoV-2 (HPIN-SARS-CoV-2) contained 159 edges and 81 nodes, including 21 viral and 60 host proteins (Fig. 5A). The significant existence of a few main gene hubs, namely, N, S, and M in the network and the attraction of a large number of low-degree nodes toward each hub showed strong evidence of control of the topological properties of the network by a few hub proteins; N with 37 degrees and S and M with 17 and 8 degrees, respectively. These viral proteins are the main hubs in the network, which regulate the network. Based on degree distribution, the viral protein N showed highest pathogenicity, followed by S and M. N is a highly conserved major structural component of SARS-CoV-2 virion involved in pathogenesis and used as a marker for diagnostic assays (28). Another structural protein, S (spike glycoprotein), attaches the virion to the cell membrane by interacting with host receptor, initiating the infection (29). The M protein, component of the viral envelope played a central role in virus morphogenesis and assembly via its interactions with other viral proteins (30). Interestingly, we found four host proteins, MYO5A, MYO5B, MYO5C and T, had a maximum interaction with viral hub proteins. MYO5A, MYO5B, and MYO5C interacting with all three proteins (N, S, and M), whereas T with two (S and M) viral hub proteins, showed a significant relationship with persistent infections caused by the SARS-CoV-2. Other host proteins showing the highest degree in the network, namely, ATP6V1G1 and RPS6, were found interacting with all the NSPs and polyprotein of ORF1a, respectively.

MYO5A, MYO5B, and MYO5C proteins are class V myosin (myosin-5) molecular motor that functions as an organelle transporter (31, 32). The presence of myosin protein played a crucial role in coronavirus assembly and budding in the infected cells (33). These cytoskeletal proteins are of importance during internalization and subsequent intracellular transport of viral proteins. It was found that inhibition of MYO5A, MYO5B, and MYO5C was efficient in blocking the internalization pathway; thus, this target can be used for the development of a new treatment for SARS-CoV-2 (34). Patients suffering from coronavirus disease 2019 (COVID-19) undergo two major conditions in the severe stage, thrombotic phenomenon and hypoxia, that are acting as silent killers (35, 36). Hypoxia, the condition where the oxygen level of the body is drastically reduced results in the elevated expression of T protein in the body (37). T protein (Brachyury/TBXT) is a transcription factor involved in regulating genes required for mesoderm formation and differentiation, thus playing an important role in pathogenesis. ATP6V1G1 (catalytic subunit of the peripheral V1 complex of vacuolar ATPase) is responsible for acidifying a variety of intracellular compartments in eukaryotic cells. It is reported that Nsp5 may cleave host ATP6V1G1, thereby modifying the host vacuole’s intracellular pH (38). RPS6 plays an important role in controlling cell growth and proliferation through the selective translation of particular classes of mRNA. Reports have shown downregulation of RPS6 during severe infections (39). The detailed functional analysis of HPIN-SARS-CoV-2 was mapped onto radiological findings from the COVID-19 severely infected patients and nonsurvivors. It was reported that the levels of fibrin-degrading proteins, fibrinogen and d-dimer protein were three- to fourfold higher than those of healthy individuals, thereby reflecting coagulation activation from infection/sepsis, cytokine storm, and impending multiple organ failure (40–43). In our network, we found 47 proteins (SUMO1, T, SMAD1-9, AGO1-4, HNRNPA1, PHB, TNN, TNR, TNXB, CXCL10, SVEP1, ANGPT1-2, ANGPT4, ANGPTL1-7, MYO5A, MYO5B, MYO5C, FGL1-2, FCN1-3, ACE2, TMPRSS2, CLEC4M, CD209, FGA, FGB, and FGG) are associated with the above etiology (Fig. 5B). We also found the interaction of SMAD family proteins and SUMO1 with N protein, which may result in inhibition of apoptosis of infected lung cells. The interactome study reveals a significant role of identified host proteins in viral budding and related symptoms of COVID-19.

### The mutation in SARS-CoV-2 proteins inhibit viral penetration into host.

**FIG 6:**
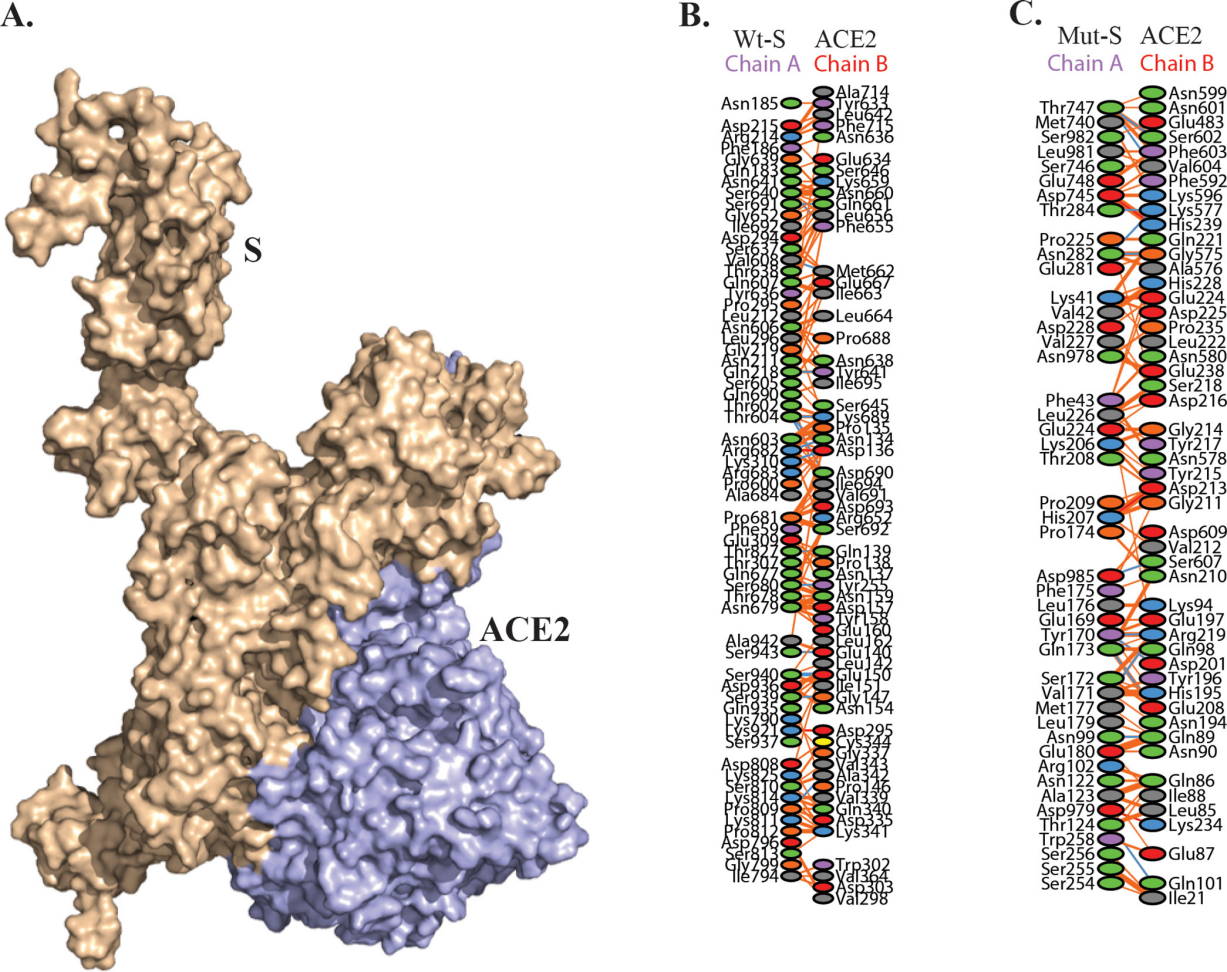
(A) *In silico* receptor-ligand docking analysis for mutated S-protein (D614G) from SARS-CoV-2 and ACE2 protein present in human. (B and C) Amino acid interactions between wild-type (Wt) and mutated spike protein with ACE2 receptor.

To validate the effect of amino acid variation (AAV), significant host protein interactions from HPIN-SARS-CoV-2 were considered for *in silico* docking studies. Docking of S-protein (wild type and mutant) with ACE2, TMPRSS2, and one of the myosin proteins (MYO5C) was analyzed. Recent studies have shown that SARS-CoV-2 uses angiotensin-converting enzyme 2 (ACE2) for entry and the serine protease TMPRSS2 for S-protein priming (44). The polyproteins (Nsp12, Nsp13, Nsp2, Nsp3, and Nsp6) of ORF1A and ORF1AB were docked with RPS6 and ATP6V1G1 host proteins. The docking results showed that mutant S-protein could not bind efficiently with ACE2 and MYO5C, whereas mutation slightly promotes the binding with TMPRSS2 (Table 3, Fig. 6, and Fig. 5B). TMPRSS2 has been detected in both nasal and bronchial epithelium by immunohistochemistry (45), reported to occur largely in alveolar epithelial type II cells which are central to SARS-CoV-2 pathogenesis (46). The wild-type S-protein forms 16 hydrogen bonds and 1,058 nonbonded contacts with ACE2, whereas the mutant protein forms 12 hydrogen bonds and 738 nonbonded contacts (Fig. 6). This result suggests that the D614G mutation in S-protein could affect viral entry into the host. Similarly, mutations present in the Nsp12, Nsp13, Nsp2, Nsp3, and Nsp6 of SARS-CoV-2 could inhibit the interaction with RPS6, but these mutations promote the binding with ATP6V1G1 except Nsp6 (L37F). RPS6 contributes to control cell growth and proliferation (47), so a loss of interaction with RPS6 could probably inhibit the production of viruses. Overall, the results of structural and interactome analyses suggest that the identified mutations (Nsp2 [T85I], Nsp3 [S1103P], Nsp6 [L37F], Nsp12 [P324L], Nsp13 [P409L and Y446C], and S [D614G]) in SARS-CoV-2 might play an important role in modifying the efficacy of viral entry and its pathogenesis. However, these observations required critical revaluation as well as experimental work to confirm the *in silico* results.

**TABLE 3:** *In silico* docking analysis of SARS-CoV-2 proteins with human proteins

SARS-CoV-2	Host protein	Wild-type score	Mutant score	Difference^*a*^
S protein	ACE2	18296	17722	574
S protein	TRMPSS2	20284	21180	−896
S protein	MYO5C	18538	17390	1148
Nsp13	RPS6	17772	15750	2022
Nsp13	ATP6V1G1	14432	20242	−5810
Nsp12	RPS6	16570	15750	820
Nsp12	ATP6V1G1	17150	20242	−3092
Nsp6	RPS6	19336	17736	1600
Nsp6	ATP6V1G1	17614	16022	1592
Nsp3	RPS6	22888	21866	1022
Nsp3	ATP6V1G1	20760	21070	−310
Nsp2	RPS6	22584	19540	3044
Nsp2	ATP6V1G1	18402	18592	−190

a Difference between the wild-type and mutant scores.

### Regulation of SARS-CoV-2 pathogenicity by CpG islands.

The genotyping analysis that we performed showed high frequency rate (20) of SNPs at the 5′ UTR region (Table 1), and a recent study also suggested that suppression of GC content could play a vital role in specific antiviral activities (28). As seen in SNP analysis, the common transitions of C→T and G→A that alter the GC content of the SARS-CoV-2 (Table 1) directed the prediction of CpG dinucleotides which are involved in silencing of transcription and downregulation of viral replication (48). In RNA viruses, CpG dinucleotides are targeted by zinc antiviral protein (ZAP), an intracellular broad-spectrum antiviral restriction factor which plays a vital role in generating innate immune response against a wide range of RNA viruses in vertebrates (49, 50). ZAP-mediated antiviral restriction has been already demonstrated against different RNA viruses, including flaviviruses, filoviruses, influenza viruses, alphaviruses, and retroviruses (51–57). ZAP directly binds to viral RNA through CCCH (Cys-Cys-Cys-His) type zinc finger motifs present at the N-terminal region and recruits RNA processing exosome for viral RNA degradation (49, 58). In association with TRIM25, ZAP binds specifically to viral RNA regions with elevated CpG dinucleotide frequencies, leading to inhibition of replication and translation of viral RNA (14, 59–61).

**FIG 7:**
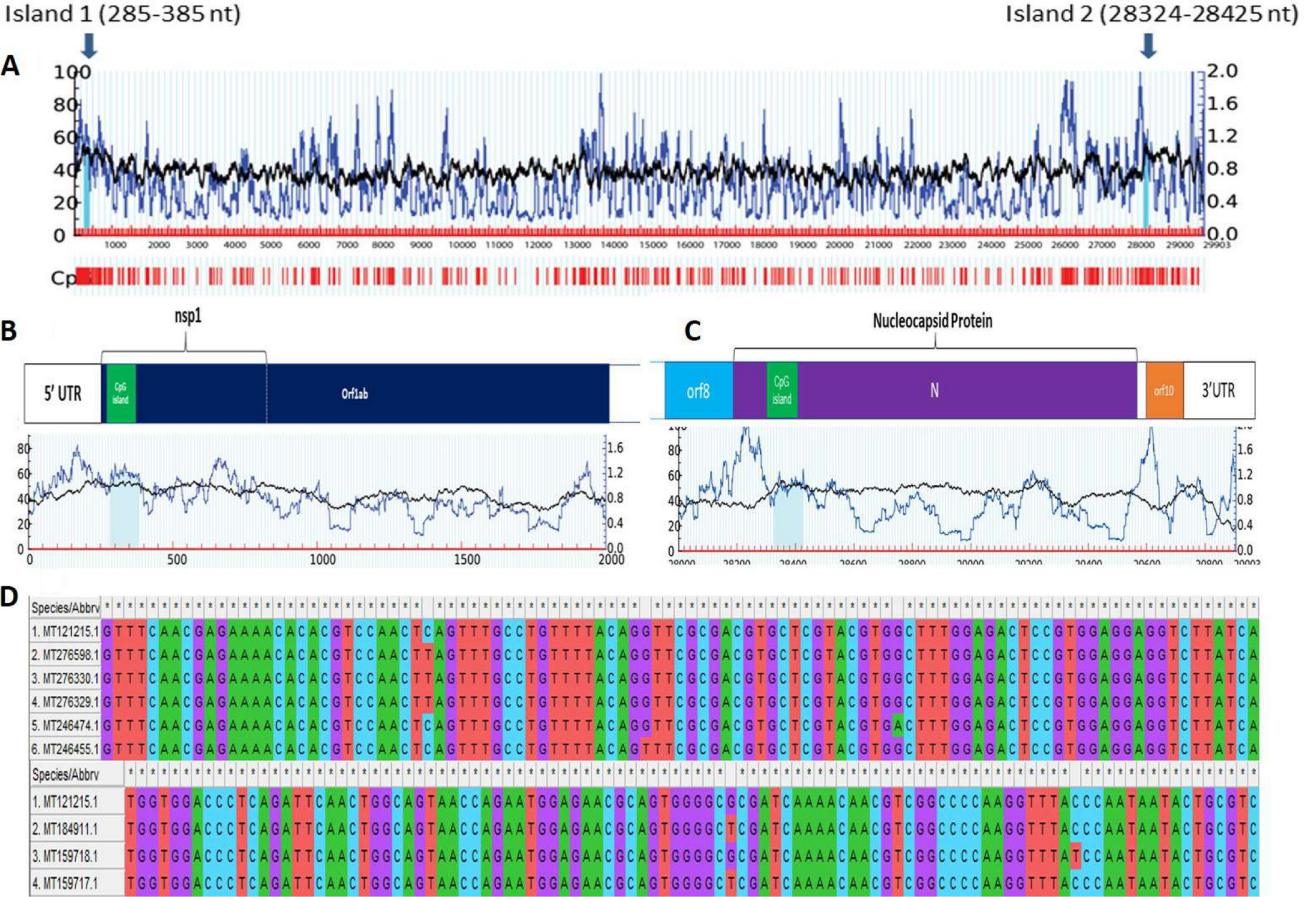
Detection of two CpG islands in Wuhan_Hu-1 complete genome sequence (accession number MT121215.1), marked by blue arrows. One of the CpG islands was found to be located toward the 5′ end of the genome, in ORF1ab. Another CpG island was found toward the 3′ end of the genome, located in ORF9 coding for N protein. nt, nucleotides.

Thus, CpG dinucleotide motif profiling and studying their importance in SARS-CoV-2 genomes was carried out. We found that CpG islands were consistently present in two regions of the genome at 285 to 385 nucleotides (101 bp) and 28,324 to 28,425 nucleotides (102 bp). The results were consistent in all 245 genomes analyzed in the present study with 100% conservancy in 237 genome sequences (Fig. 7).

In the remaining eight genomes, five genomes (MT246474.1 [G-to-A substitution at position 354 with respect to the reference genome], MT276329.1, MT276330.1, and MT276598.1 [C-to-T substitution at position 313], and MT246455.1 [G-to-T substitution at position 332]) showed point mutations in the 5′ CpG island, whereas three genomes (MT159718.1 [C-to-T substitution at position 2840] and MT159717.1 and MT184911.1 [G-to-T substitution at position 28378]) showed point mutations in the 3′ CpG end. Interestingly, all these sequences belong to the United States. On further locating CpG island positions with respect to proteins, it was found that these two CpG islands were located at two prime locations within the genome, one in Nsp1, and another within N protein. Previously, it was reported that both the proteins interacted with the 5′ UTR region playing crucial roles in viral replication and gene expression (4, 62, 63). The most pivotal role of the N protein revolves around encapsulation of viral genome RNA (gRNA) which leads to formation of ribonucleoprotein complex (RNP), which is a vital step in assembly of viral particles (64).

Nsp1 protein in coronaviruses plays a regulatory role in transcription and viral replication (64). It is known to interact with 5′ UTR of host cell mRNA to induce its endonucleolytic cleavage (65, 66), thus inhibiting host gene expression (67). It also plays an important role in blocking interferon (IFN)-dependent antiviral signaling pathways leading to dysregulation of host immune system (68–70). CpG sites can be targeted by zinc finger antiviral proteins which can mediate antiviral restriction through CpG motif detection (51, 56, 57). Apart from this, CpG oligodeoxynucleotides (ODNs) are known to act as adjuvants and are already established as a potent stimulator for host immune system (71–74). Moreover, recent studies conducted on influenza A and Zika virus genome has shown that by increasing the CpG dinucleotides in viral genome, impairment of viral infection is observed (75, 76). Our result showed that the presence of conserved CpG islands in Nsp1 and N protein across all genomes of SARS-CoV-2 indicated their role in pathogenesis and can be targeted by zinc finger antiviral proteins or exploited to design CpG-recoded vaccines.

### Conclusions.

The genomic and proteomic survey of SARS-CoV-2 strains reported from subsets of populations of different countries reflected global transmission during the outbreak of COVID-19. The viral phylogenetic network with five clades (a to e) provided a landscape of the current stage of epidemic where major divergence was observed in U.S. strains. From this, we propose genotypes linked to geographic clades in which signature SNPs can be used to track and monitor the epidemic. Demarcation of co-mutation in the SARS-CoV-2 strains by assessing co-mutations also highlighted the evolutionary relationships among the viral proteins. Our results suggested that co-mutations are indicative of AAV-based induced pathogenicity leading to multiple mutations embedded in a few genomes. It was also seen that just increasing the genomic sample size by 50 times did not led to prediction of significant mutations or co-mutations that were leading to strain variation in SARS-CoV-2 virus. Thus, sample size of SARS-CoV-2 genome does not have a direct relation with variation to be predicted in amino acids. However, co-mutations are still in evolutionary process, and more combinations can be predicted with a large data set. High-frequency AAV mutations were present in the critical proteins, including the Nsp2, Nsp3, Nsp6, Nsp12, Nsp13, S, Orf3a, and Orf8 which could be considered for designing a vaccine. Comparative analysis of proteins from wild and mutated strains showed positive selection of mutation in Nsp3 but not in the rest of the mutants. The HPI model can be used as the fundamental basis for the structure-guided pathogenesis process inside the host cell. The interactome study showed MYO-5 proteins as a key host partner and highlighted the key role of N, S, and M viral proteins for conferring SARS-CoV-2 pathogenicity. The mutation in the S-protein could affect the viral entry by loose binding with ACE2. The presence of CpG dinucleotides in N and Nsp1 protein could play a critical role in pathogenesis regulation. Based on our multi-omics approach, genomics, proteomics, interactomics, and systems and structural biology provided an opportunity for better understanding of COVID-19 strains and its mutational variants.

## MATERIALS AND METHODS

### Selection of genomes, annotations, and phylogeny construction.

Publicly available genomes of SARS-CoV-2 viruses were obtained from the NCBI database (https://www.ncbi.nlm.nih.gov/genbank/sars-cov-2-seqs/). Until 31 March 2020, only 447 SARS-CoV-2 genomes (see Data Set S1, sheet 1, in the supplemental material) were available in the databases (supplemental material). The data were screened for unwanted ambiguous bases using N-analysis program, based on which 245 (Data Set S1, sheet 2) complete and clean genomes of SARS-CoV-2 were selected for further analysis (supplemental material). A manually annotated reference database was generated using GenBank file of severe acute respiratory syndrome coronavirus 2 isolate SARS-CoV-2/SH01/human/2020/CHN (accession number MT121215.1) and open reading frames (ORFs) were predicted against the formatted database using prokka (-gcode 1) (77). Genomic sequences included in the analysis belong to different countries, namely, the United States (168), China (53), Pakistan (2), Australia (1), Brazil (1), Finland (1), India (2), Israel (2), Japan (5), Vietnam (2), Nepal (1), Peru (1), South Korea (1), Spain (1), and Sweden (1). Whole-genome nucleotide and protein sequences were aligned using mafft (78) at 1,000 iterations. The alignments so obtained were processed for phylogeny construction using BioEdit software (79). The nucleotide-based phylogeny was annotated and visualized on the iTOL server (80), while amino acid-based phylogeny was visualized and annotated using GrapeTree (81).

10.1128/mSystems.00030-21.2 DATA SET S1 Summary of the SARS-CoV-2 genomes with isolated geographical location selected for comparative genomics analysis. Sheet 1, 447 genomes; sheet 2, 245 genomes; sheet 3, 18,775 genomes; sheet 4, 12,299 genomes. Download Data Set S1, XLSX file, 0.5 MB. Copyright © 2021 Gupta et al. 2021 Gupta et al. https://creativecommons.org/licenses/by/4.0/ This content is distributed under the terms of the Creative Commons Attribution 4.0 International license.

10.1128/mSystems.00030-21.3 DATA SET S2 Summary of the mutations identified from the 245 SARS-CoV-2 genomes and selected co-mutations. Download Data Set S2, XLSX file, 0.02 MB. Copyright © 2021 Gupta et al. 2021 Gupta et al. https://creativecommons.org/licenses/by/4.0/ This content is distributed under the terms of the Creative Commons Attribution 4.0 International license.

10.1128/mSystems.00030-21.4 DATA SET S3 Summary of the mutations identified from the 12,299 SARS-CoV-2 genomes. Download Data Set S3, XLSX file, 0.6 MB. Copyright © 2021 Gupta et al. 2021 Gupta et al. https://creativecommons.org/licenses/by/4.0/ This content is distributed under the terms of the Creative Commons Attribution 4.0 International license.

### Genotyping based on SNP/AAV.

To detect nucleotide and amino acid variations (AAV) among 245 genomes of SARS-CoV-2, sequence alignment of nucleotide and amino acid, respectively, were performed against the reference genome. The nucleotide and amino acid changes were calculated as point variations and recorded. The interpolation and visualization were plotted using computer programs in Python. Co-mutations were predicted, and clustering was performed using MicroReact (82). For validation we selected 18,775 (Data Set S1, sheet 3) complete genomes available in the NCBI virus database (see “Data availability” below) last accessed in September 2020. After the genomes containing sequencing errors and unidentified base pairs “N.” were removed, the remaining 12,299 genomes were used (Data Set S1, sheet 4).

### Data and computer programs.

The genomic analytics is performed using programs in Python and Biopython libraries (83). The computer programs and the updated SNP profiles of SARS-CoV-2 isolates are available upon request.

### Construction of the host-pathogen interaction network of SARS-CoV-2.

The interactions between viral and host proteins are responsible for all aspects of the viral life cycle; from infection of the host cell to replication of the viral genome and assembly of new viral particles (84). To find the host-pathogen interaction (HPI), we subjected SARS-CoV-2 proteins sequence to host-pathogen interaction databases such as Viruses STRING v10.5 (85) and HPIDB3.0 (86) to predict their direct interaction with humans as the principal host. In these databases, the virus-host interaction was imported from different PPI databases like MintAct (87), IntAct (87), HPIDB (86), and VirusMentha (88). It searches protein sequences using BLASTP to retrieve homologous host/pathogen sequences. For high-throughput analysis, it searches multiple protein sequences at a time using BLASTp and obtains results in tabular and sequence alignment formats (89). The HPI network was constructed and visualized using Cytoscape v3.7.2 (90). It is an open-source software platform for visualizing molecular interaction networks which involve various biological pathways and integrating these networks with annotations, gene expression profiles, and other state data. In the constructed network, proteins with the highest degrees, which interact with several other signaling proteins in the network, indicate a key regulatory role as a hub. In our study, using Network Analyzer (91), a plugin of Cytoscape v3.7.2, we identified the hub protein. Further, the human proteins interacting with individual viral proteins were subjected to functional annotation. Gene ontology (GO) analysis was performed using ClueGo (92), selecting the Kyoto Encyclopedia of Genes and Genomes (KEGG) (93), Gene Ontology—biological function database, and Reactome Pathways (94) databases. The ClueGo parameters were as follows: Go Term Fusion selected; pathways or terms of the associated genes, ranked based on the *P* value corrected with Bonferroni stepdown (*P* values of <0.05); GO tree interval, all levels; GO term minimum number of genes, 3; threshold, 4% of genes per pathway; kappa score, 0.42. Gene ontology terms are presented as nodes and clustered together based on the similarity of genes corresponding to each term or pathway.

### Computational structural analysis on wild-type and mutant SARS-CoV-2 proteins.

SARS-CoV-2 protein sequences were retrieved from the NCBI genome database, and pairwise sequence alignment of wild-type and mutant proteins were carried out by the Clustal Omega tool (95). The wild-type and mutant homology model of S-protein, Nsp12, and Nsp13 were constructed using the SWISSMODEL (96), whereas the three-dimensional (3D) structure of ORF8, ORF3A, Nsp2, Nsp3, and Nsp6 were predicted using Phyre2 server (97). The crucial host proteins (TMPRSS2, RPS6, ATP6V1G1, and MYO5C) 3D structures were generated using the SWISSMODEL and ACE2 structure retrieved from the PDB database (PDB identifier [ID] 6M17). These structures were energy minimized by the Chiron energy minimization server (98). The effect of the mutation was analyzed using HOPE (99) and I-mutant (100). The I-mutant method allows us to predict the stability of the protein due to mutation. The docking studies for wild and mutant SARS-CoV-2 proteins with host proteins were carried out using PatchDock Server (101). Structural visualizations and analysis were carried out using pyMOL2.3.5 (102).

### Analysis of CpG regions.

SARS-CoV-2 genomes were analyzed for the presence of CpG regions. To locate the CpG regions, meth primer 2.0 (http://www.urogene.org/methprimer2/) and the CpG Plot (http://www.ebi.ac.uk/Tools/emboss/cpgplot/) programs were used, although some variations were found in both the programs. Both the programs were run on default parameters of a sequence window longer than 100 bp, a GC content of ≥50%, and an observed/expected CpG dinucleotide ratio of ≥0.60. The presence of common CpG islands was confirmed by performing BLAST using the above reference strain.

### Data availability.

Complete set of sequences for full-length genomes and proteomes of SARS CoV-2 virus used in the study are available at https://www.ncbi.nlm.nih.gov/labs/virus/vssi/#/virus?SeqType_s=Nucleotide%20seafood=%20market=%20pneumonia=%20virus,=%20taxid:2697049=&VirusLineage_ss=Severe%20acute%20respiratory%20syndrome%20coronavirus%202%20(SARS-CoV-2),%20taxid:2697049.
