# Supplementary figures and images for: Comparative Genomics and Integrated Network Approach Unveiled Undirected Phylogeny Patterns, Co-mutational Hot Spots, Functional Cross Talk, and Regulatory Interactions in SARS-CoV-2
Source: mSystems. 2021 Feb 23;6(1):e00030-21. doi: 10.1128/mSystems.00030-21 (PMC8573956; doi:10.1128/mSystems.00030-21)

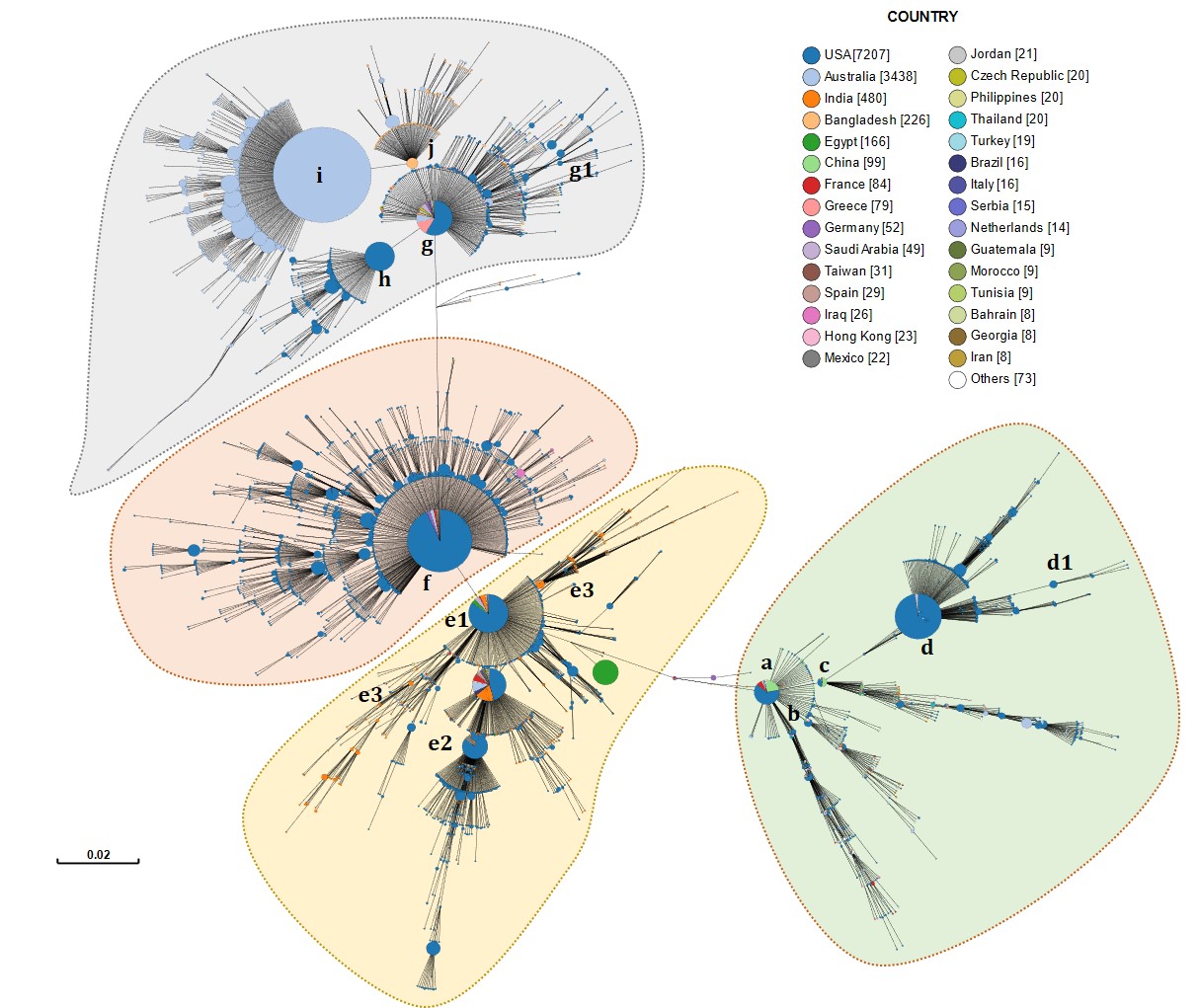

Supplement: FIG S1 [file msystems.00030-21-sf001.jpg]
